# Tacticity-Dependent Interchain Interactions of Poly(*N*-Isopropylacrylamide) in Water: Toward the Molecular Dynamics Simulation of a Thermoresponsive Microgel

**DOI:** 10.3390/gels3020013

**Published:** 2017-04-19

**Authors:** Gaio Paradossi, Ester Chiessi

**Affiliations:** Department of Chemical Sciences and Technologies, University of Rome Tor Vergata, Via della Ricerca Scientifica I, 00133 Roma, Italy; paradossi@stc.uniroma2.it

**Keywords:** PNIPAM, tacticity, LCST, volume phase transition temperature (VPTT), hydrogels, polymer network

## Abstract

The discovery that the lower critical solution temperature (LCST) of poly(*N*-Isopropylacrylamide) (PNIPAM) in water is affected by the tacticity opens the perspective to tune the volume phase transition temperature of PNIPAM microgels by changing the content of meso dyads in the polymer network. The increased hydrophobicity of isotactic-rich PNIPAM originates from self-assembly processes in aqueous solutions also below the LCST. The present work aims to detect the characteristics of the pair interaction between polymer chains, occurring in a concentration regime close to the chain overlap concentration, by comparing atactic and isotactic-rich PNIPAM solutions. Using atomistic molecular dynamics simulations, we successfully modelled the increased association ability of the meso-dyad-rich polymer in water below the LCST, and gain information on the features of the interchain junctions as a function of tacticity. Simulations carried out above the LCST display the PNIPAM transition to the insoluble state and do not detect a relevant influence of stereochemistry on the structure of the polymer ensemble. The results obtained at 323 K provide an estimate of the swelling ratio of non-stereocontrolled PNIPAM microgels which is in agreement with experimental findings for microgels prepared with low cross-linker/monomer feed ratios. This study represents the first step toward the atomistic modelling of PNIPAM microgels with a controlled tacticity.

## 1. Introduction

Polymer microgels based on poly(*N*-isopropylacrylamide) (PNIPAM) are the most famous paradigm of stimuli-responsive soft microdevices [1]. The thermal sensitivity of PNIPAM microgels comes from the lower critical solution temperature (LCST) behaviour of PNIPAM aqueous solutions, that, in hydrated polymer networks, causes a volume phase transition (VPT) between a swollen state and a shrunk state at a VPT temperature similar to the LCST of PNIPAM in water. The temperature trigger was the first element exploited for the responsivity of PNIPAM microgels and it opens the perspective toward applications in biomedicine, since the VPT temperature, about 32 °C, is close to the physiological temperature. Later, several variants of PNIPAM based microgel were proposed by introducing different polymers in the network to add other responsivity factors, such as pH and ionic strength [2,3].

Tuning the value of the VPT temperature (VPTT) to adapt the PNIPAM microgels to specific applications is typically accomplished by including more, or less, hydrophobic residues as compared to PNIPAM, with a corresponding lowering, or increase, of the VPTT. Other options to change the VPTT are to modify the composition of the solution that swells the microgel, for example by using mixed solvents [4] or high ionic strength [5]. All these methods alter the chemical features of the original polymer matrix and the microgel biocompatibility with possible consequences on application. A novel alternative, which does not contaminate the PNIPAM scaffold or the swelling medium of the microgel, is to focus on the tacticity-dependent hydrophobicity of this polymer. It has been observed that stereoisomers with an increased content of racemo dyads, namely syndiotactic-rich PNIPAMs, display a LCST higher as compared to the LCST of the atactic PNIPAM [6,7]. On the contrary, stereoisomers with a higher content of meso dyads, namely isotactic-rich PNIPAMs, show a decrease of the LCST in aqueous solution [8]. The LCST modulation caused by a variation of stereochemistry can span several degrees, coming from the LCST of 17 °C for PNIPAMs with degree of polymerization (DP) of about 300 and percentage of meso dyad,* m*, of 66% [9] up to the LCST of about 37 °C for syndiotactic-rich PNIPAMs with 17% < *m* < 25% [6]. In particular, PNIPAM chains with *m* values higher than approximately 70% are not soluble in water [9,10]. These results imply that to include fragments of isotactic-rich PNIPAM in the atactic polymer network of the microgel has the same effect as to insert a hydrophobic component.

Chemically cross-linked monolithic hydrogels of meso-diad-rich PNIPAMs were recently investigated [10,11]. Such hydrogels were prepared with a feed molar ratio of cross-linker to repeating unit, *N,N*′-methylenebisacrylamide/*N*-isopropylacrylamide, of 1/27, corresponding to an average chain length between crosslinks of about 14 residues. As compared to the corresponding non-stereocontrolled hydrogel, these systems display lower swelling ratios and higher deswelling rates below and above the VPTT, respectively, in agreement with the increased hydrophobicity of the polymer network. 

The reduced water affinity of isotactic-rich PNIPAMs induces different modalities of self-association of the linear homopolymer in aqueous solution, depending on the distribution of the isotactic tracts along the chain. When the isotactic domains are randomly distributed, stable intermolecular polymer chain junctions [12] and hydrogel phases [13] form in water below the LCST. This is not the case with the corresponding atactic polymers [12,13]. In particular, the work of Nakano et al. [13] represents the first instance of thermoreversible gelation in networks totally composed by PNIPAM. It is noteworthy that the systems in [12] and [13] show the transition to the water-insoluble state with the typical phase separation by a further increase of temperature. When the isotactic domains are localized in specific regions of the chain, as in A–B–A stereoblock copolymers, with A and B blocks consisting of isotactic-rich and atactic PNIPAM sequences, respectively, the self-association leads to hydrogel phases [14] or nano-aggregates [15] depending on the polymer concentration. Copolymers composed of A and B blocks having DP of about 18 and 250, respectively, with A blocks having a meso dyad content of 70%–80%, form physically cross-linked hydrogels at room temperature for polymer concentrations above 4.5% (*w*/*w*). Differently, core-shell nanoparticles are spontaneously formed for polymer concentrations below 0.5% (*w*/*w*), where the isotactic blocks are confined in the core [15]. It is noteworthy that the copolymer precipitation is detected for such hydrogels above the PNIPAM cloud point of 32 °C [14], whilst the nanoparticle dispersions display a high colloidal stability even at 45 °C, well above the LCST, with only a slight turbidity [15]. The last result is ascribed to the increased intra- and interchain hydrogen bonding of the isotactic PNIPAM within the micelle core, that produces constraints on the looped atactic blocks in the shell. In such conditions, the collapse of the copolymer chain, made mainly of atactic PNIPAM, is hindered and the colloidal suspension is stabilized [15]. The chain collapse is indeed the first step of the temperature-induced phase segregation of PNIPAM in aqueous environment.

Because of the self-association ability of isotactic-rich PNIPAM, the amount of meso dyad is a factor able to affect the topology of the polymer network in nanogels or microgels based on such stereoisomers. Indeed, physical junctions between isotactic regions will develop in addition to the chemical cross-links, whose extent can be controlled by the cross-linker/monomer feed ratio used in the synthesis. Therefore, the microgel will show features common to chemical and physical hydrogels, by increasing the versatility of such systems.

In this context, the purpose of the present work is to investigate, at a molecular level, the characteristics of the inter-chain interaction of PNIPAM in aqueous solution as a function of tacticity, by means of atomistic molecular dynamics (MD) simulations. This method was successfully applied to study the temperature-induced coil-to-globule transition of PNIPAM at infinite dilution for model chains with different stereochemistries [16,17,18,19]. Here, we aim to highlight the differences in the association behaviour of atactic PNIPAM and an isotactic PNIPAM with *m* of 59%, both below and above the LCST. The atactic chain has an *m* of 45%, since it is shown that this value is obtained for PNIPAM synthesized with non-stereoselective methods [20,21]. The distribution of meso and racemo dyads in the stereoisomer is assumed as Bernoullian. The model is formed by two linear chains, each of 30 residues and with equal stereochemistry, in water at a concentration close to the chain overlap concentration, C*. In these conditions, the salient characteristics of the inter-chain interaction as a function of tacticity are enhanced, as compared to a more concentrated solution where non-specific aggregations can occur. Moreover, this model represents the precursory processes of association, focusing on the pair interaction between chains. The stereochemistry of the chains was selected on the basis of the work of Nishi et al. [22], where the solution behaviour of stereoisomers with *m* 45% and 58% is described. In such experimental study, a molecular interpretation of the LCST lowering of the isotactic-rich PNIPAM is proposed, by attributing this result to the increased inter-chain association ability of the stereocontrolled PNIPAM. MD simulations are carried out at 283 K, where the aqueous solution of both stereoisomers is thermodynamically stable [22], and at 323 K, above the LCST, in the time window of 210 ns. The results of simulations at 283 K show the association of the isotactic-rich stereoisomers sustained by hydrophobic interactions, in agreement with experimental findings. At 323 K, the solution models display the coil-to-globule transition of chains, concerted with aggregation, irrespective of stereochemistry. From the trajectory analysis, we reveal the details of the inter-chain junctions below the LCST as a function of tacticity and we provide an estimate of the swelling ratio of non-stereocontrolled PNIPAM microgels.

## 2. Results and Discussion

### 2.1. Below the LCST

**Figure 1:**
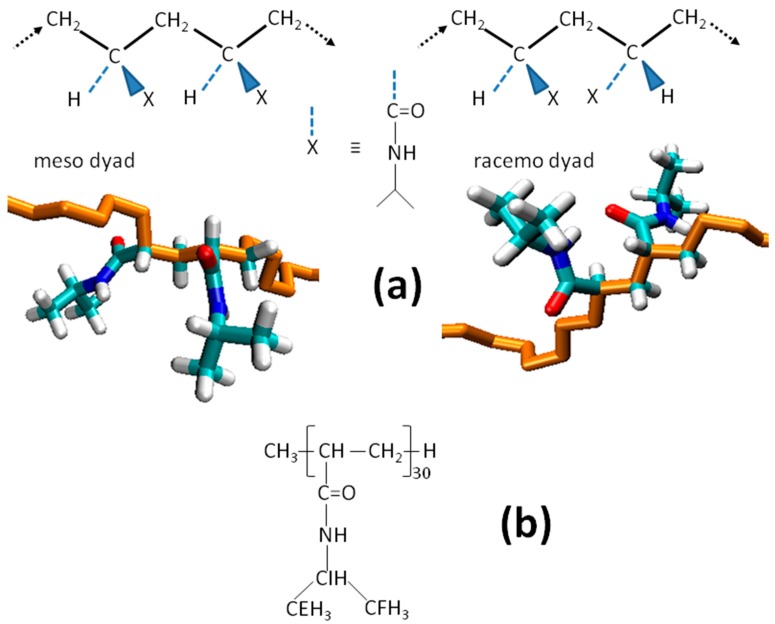
(**a**) Illustration of the meso and racemo dyads within the poly(*N*-Isopropylacrylamide) (PNIPAM) chain. (**b**) Chemical structure of the stereoisomers and nomenclature of side chain atoms.

Figure 1a illustrates the stereochemistry of the meso and racemo dyads within the PNIPAM chain. The dyads sequence of the atactic and isotactic-rich stereoisomers, containing 45% and 59% of meso dyads, respectively, is reported in Section 4. To directly monitor the inter-chain interactions, we considered the contacts between PNIPAM atoms belonging to different chains. The nomenclature of atoms is reported in Figure 1b. The analysis included the contacts formed by the atoms pairs: N(A)–O(B); N(B)–O(A); CE_CF(A)–CE_CF(B); CI(A)–CI(B); CE_CF(A)–CI(B); CI(A)–CE_CF(B), where the letters A and B indicate the chain. The presence of the X–Y contact is detected on the basis of a cutoff distance equal to the first minimum distance of the corresponding inter-chain radial distribution functions. The cutoff distance values are reported in Section 4. The contacts between oxygen and nitrogen atoms are related to dipolar or HB interactions, whilst the other contacts are accounted as hydrophobic or van der Waals interactions.

**Figure 2:**
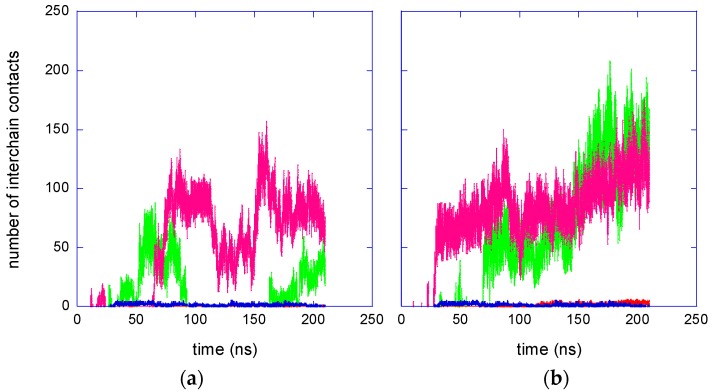
Number of inter-chain contacts in the PNIPAM solutions. Hydrophobic contacts for the atactic and isotactic-rich stereoisomers are displayed in pink and green, respectively. Contacts between N and O atoms for the atactic and isotactic-rich stereoisomers are displayed in red and blue, respectively: (**a**) *T* = 283 K; (**b**) *T* = 323 K.

The time behaviour of the number of inter-chain contacts in the atactic and isotactic-rich PNIPAM solutions at 283 K is shown in Figure 2a. Polar and hydrophobic contacts are individually displayed.

Figure 2a shows that, below the LCST, several contacts involving hydrophobic atoms develop between the chains of the isotactic-rich PNIPAM during the equilibration phase and that inter-chain contacts persist in the following trajectory. A much smaller number of hydrophobic contacts is detected for the atactic stereoisomer, and, in particular, these chains do not form any contact throughout about half of the production run. Additionally, the contacts between oxygen and nitrogen atoms show the same difference between stereoisomers. It is noteworthy that the N–O contacts are less frequent as compared to hydrophobic contacts, irrespective of stereochemistry, also if the lower statistical probability of polar contacts is taken into account. This result is explained by the hydration of the amide moiety, preventing association of polar groups at 283 K.

**Figure 3:**
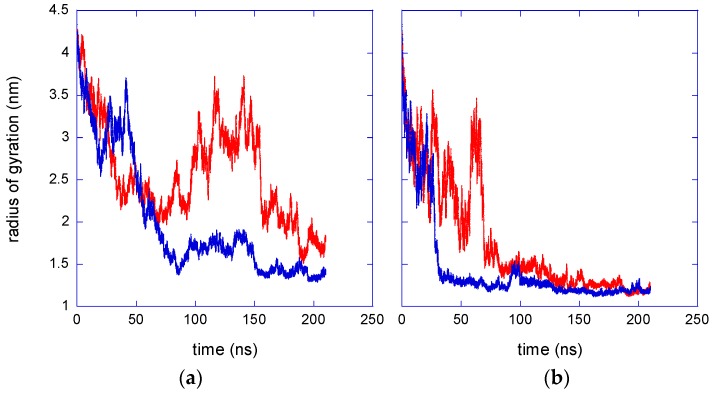
Time behaviour of the radius of gyration of the two-chain ensemble. Red and blue curves refer to atactic and isotactic-rich PNIPAM, respectively. (**a**) *T* = 283 K; (**b**) *T* = 323 K.

The formation of a stable aggregate can cause a reduction of the size of the two-chain ensemble. This effect is indeed detected in the simulations, as shown by the time behaviour of the overall radius of gyration at 283 K, reported in Figure 3a.

The correlation between the number of inter-chain contacts and the size of the PNIPAM cluster is evident by the comparison of Figure 2a and Figure 3a. The formation of a stable aggregate in the isotactic-rich PNIPAM solution, visible in Figure 2a at about 80 ns, corresponds, in Figure 3a, to a severe size reduction. Moreover, in the last 40 ns trajectory, the chains are in contact both in the atactic and isotactic-rich PNIPAM solution, but in the last system the number of contacts is about triple. Consequently, in the same time interval, the average radius of gyration of the isotactic-rich PNIPAM ensemble is 1.40 ± 0.05 nm, sensibly lower as compared to the value of 1.8 ± 0.2 nm, obtained for the atactic polymer.

**Figure 4:**
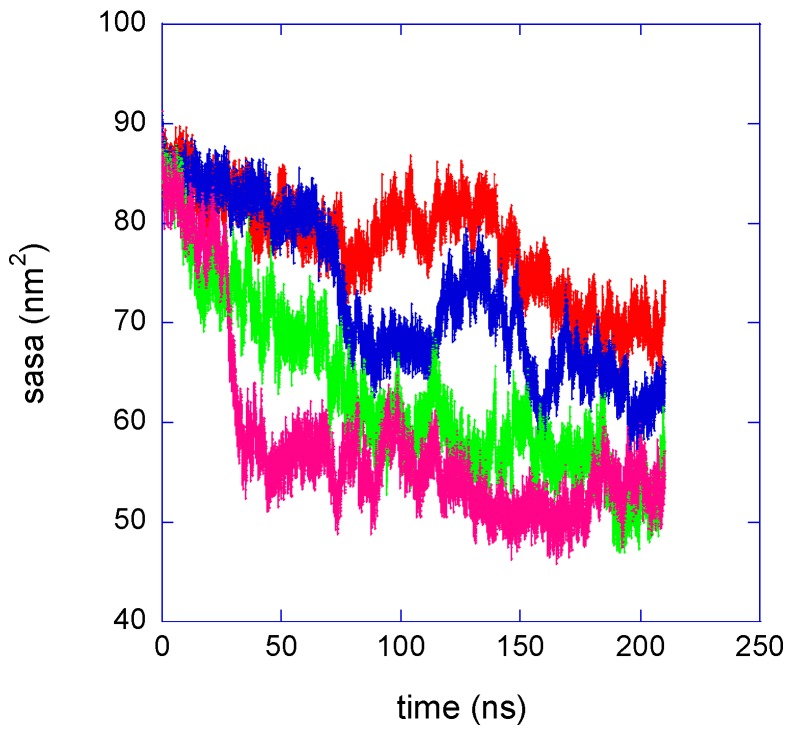
Time behaviour of the solvent surface accessible area of the two-chain ensemble. Red and blue curves refer to atactic and isotactic-rich PNIPAM, respectively, at *T* = 283 K. Green and pink curves refer to atactic and isotactic-rich PNIPAM, respectively, at *T* = 323 K.

The solvent accessible surface area, sasa, of a solute molecule is the surface of closest approach of the centres of solvent molecules where both solute and solvent are represented by hard spheres. Computationally, this surface is defined as the van der Waals envelope of the solute molecule expanded by the radius of the solvent sphere about each solute atom centre [23]. The association of PNIPAM chains provokes a decrease of the solvent surface accessible area, as shown in Figure 4.

The reduction of the solute–water interface is the driving force of the association of hydrophobic moieties in aqueous environment, for the entropy gain of releasing water molecules from the hydration shell. In agreement with the experimentally observed hydrophobic character of isotactic-rich PNIPAMs, at 283 K the meso-dyad-rich polymer ensemble displays smaller sasa values as compared to the atactic one, Figure 4. For both systems, different chain conformations are populated during the production run, corresponding to variable sasa values. However, a sasa difference of ≈5 nm^2^ between atactic and isotactic-rich PNIPAM ensemble can be estimated from the behaviours at 283 K in Figure 4.

The difference between the sasa values of the atactic and isotactic-rich PNIPAMs corresponds to a gain of free energy of hydration for the isotactic-rich polymer, caused by the higher inter-chain association. Assuming a free energy decrease for the reduction of the sasa of ≈10 kJ·mol^−1^·nm^2^, deduced from solubility data of hydrocarbons in water [24], a free energy difference of ≈50 kJ/mol is roughly estimated. To discuss this datum, we consider the results of an experimental and molecular modelling study of the PNIPAM dimer diastereomers in aqueous solution [25]. In such work, the difference between the free energy of hydration of the meso and racemo dimers, ΔG_r→m_, at 298 K is determined as equal to 1.2 kJ/mol. The stereoisomers of the present work are 30-mers with percentages of meso dyads of 45% and 59%, so the two-chain ensemble of the isotactic-rich PNIPAM contains an extra number of eight meso dyads, as compared to the atactic PNIPAM. With the value of ΔG_r→m_ in [25], the difference of hydration free energy between the atactic and isotactic-rich PNIPAM ensembles results equal to 10 kJ/mol at 298 K. The last value can be compared with the estimate of 50 kJ/mol from these simulations, that represents the gain in free energy of the meso-dyad-rich PNIPAM deriving from the greater chains’ association. By considering that simulations are carried out at 283 K and that in this estimate we are assuming that hydration free energy of the dimer is the same as for the dyad inserted in the polymer chain, the sasa behaviour observed in the simulations is compatible with the findings on PNIPAM dimer solubility. The increased inter-chain association of the isotactic-rich system allows for a free energy decrease able to compensate the lower water affinity of this stereoisomer, as compared to the atactic polymer. 

**Figure 5:**
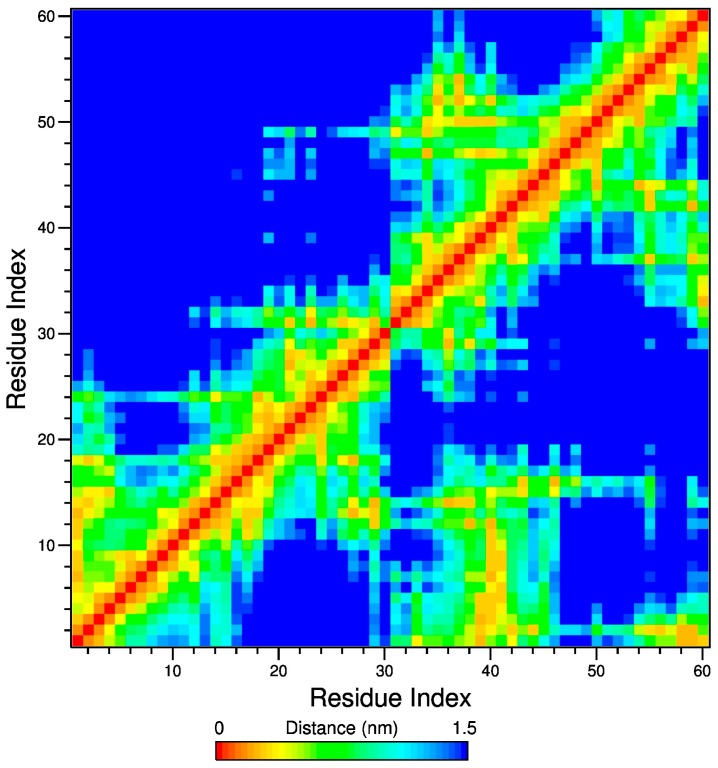
Inter-residues contact map at 283 K in the interval 200–202 ns. The top-left and bottom-right regions across the diagonal refer to atactic and isotactic-rich PNIPAM, respectively.

To highlight the inter-residue connectivity within the PNIPAM ensemble, both intra- and inter-molecularly, we calculated the matrixes of the average minimum distance between the residues during the trajectory. An example of such matrixes is shown in Figure 5, displaying the matrix at 283 K in the 200–202 ns trajectory interval. In this representation, using a colour code for the distance value, orange-yellow spots indicate a pair of residues in contact. Residues 1–30 and 31–60 form the first and second chain, A and B, respectively, therefore, by considering the four quadrants forming the map, the intrachain contacts are visible in the bottom left square for chain A, and in the top right square for chain B, whilst the inter-chain contacts are displayed in the bottom right and top left squares. 

For the sake of comparison between stereoisomers, in Figure 5, the top region above the map diagonal shows the result obtained for the atactic PNIPAM and the bottom region the result for the isotactic-rich polymer. Figure 5 and all of the maps calculated in the production run highlight that the inter-chain contacts are much more numerous and frequent for the system with the higher content of meso dyads, in agreement with the behaviours of Figure 2a. However, analysis of Figure 5 reveals that the distribution and extent of intramolecular contacts are similar for the two stereoisomers. This peculiar result indicates that the more hydrophobic character of the isotactic-rich PNIPAM affects preferentially the inter-chain connectivity, whilst the intramolecular interactions are minimally perturbed by changes of tacticity. Such a simulation finding is in agreement with the experimental study of Nishi et al. [22] on stereocontrolled PNIPAMs with equal dyad composition as our models. Polymer chains display the same chain size, irrespective of stereochemistry, in diluted aqueous solution below the LCST, that implies a similar intra-chain connectivity [22]. To confirm this result, we analyzed the end-to-end distance, r, of the chains during the simulation and the average values are summarized in Table 1. The differences between the r values of the atactic and isotactic-rich chains are within errors.

**Figure 6:**
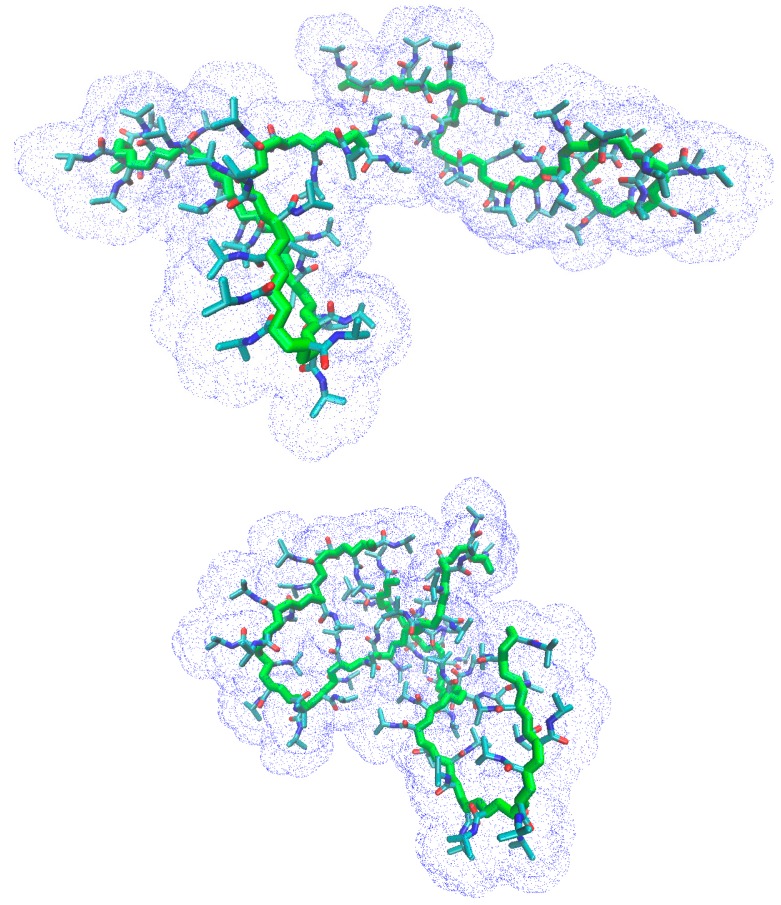
Snapshot at 202 ns of the trajectory of the atactic (**top**) and isotactic-rich (**bottom**) PNIPAM ensemble at 283 K. Water and hydrogen atoms are omitted. The blue dots display the solvent accessible surface.

Figure 6 shows the snapshots at 202 ns of the trajectory at 283 K for the atactic and isotactic-rich PNIPAM, to support the interpretation of Figure 5.

**Figure 7:**
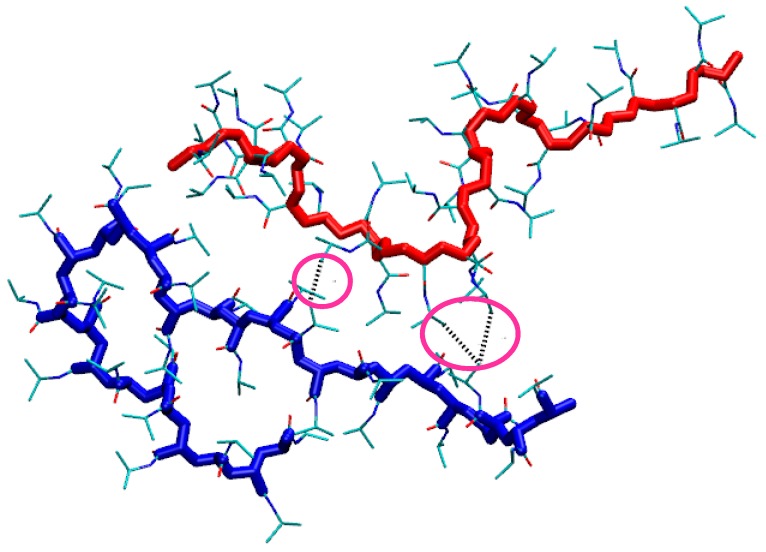
Snapshot at about 130 ns of the trajectory of the isotactic-rich PNIPAM at 283 K. Water and hydrogen atoms are omitted. The backbone atoms are coloured in red and blue for chain A and B, respectively. The pink circles highlight the junctions.

The stability of the physically crosslinked hydrogels of isotactic-rich PNIPAM [13,26] and the self-assembly of stereoblock PNIPAM copolymers [14,15] is founded on the formation of interchain junctions, whose features influence the topology of the macromolecular network in hydrogels and the modality of association in polymer micelles. For a polymer hydrogel, the degree of crosslinking (DC) is a key parameter, determining porosity, rheological and osmotic features of hydrogel matrixes. In a schematic representation of the network, the DC values depend on the number of junctions per chain (NJ) and on the number of residues involved in the single junction (LJ). With the aim to characterize in detail the association pattern, we analyzed these features in the PNIPAM ensembles of the simulations. For each time frame, the number of junctions formed by the chain, A or B, with the other chain, B or A, referred to as NJ_A_(t) and NJ_B_(t), respectively, was monitored, by considering as a junction or a single residue or a group of adjacent residues of the chain involved in interchain contacts with the other chain. The length (in residues) of each junction was determined and the mean junction length at each time frame, LJ_mean_(t), was calculated for both chains. The number of residues of chain A, or B, involved in contacts with chain B, or A, referred to as NR_A_(t), or NR_B_(t), respectively, is equal to the product NJ_A_(t)* LJ_mean,A_(t), or NJ_B_(t)* LJ_mean,B_(t). An example of these structural parameters of the interchain association is illustrated in Figure 7. The pink circles highlight the junctions, where the first junction (from the left) involves one residue of A and one residue of B; the other junction involves two adjacent residues of A and one residue of B. Therefore, in the snapshot of Figure 7: NR_A_ = 3, NJ_A_ = 2, LJ_mean,A_ = 1.5; NR_B_ = 2, NJ_B_ = 2, LJ_mean,B_ = 1.

The results of the junctions analysis during the production run are reported in Table 1. Irrespective of stereochemistry, the interaction between the two chains can be considered as symmetrical, since the values related to contacts of chain A with chain B are equal, within errors, to those related to contacts of B with A. The average values of NR obtained at 283 K for the atactic chains are significantly lower than the corresponding values of the isotactic-rich stereoisomers. Data of NR in Table 1 shows that about 10% of residues of the atactic stereoisomer are implicated when chains come into contact, whilst about 30% of residues participate in interchain contacts for the isotactic-rich stereoisomer. Therefore, not only is the probability of the atactic chain to form junctions at 283 K about half as compared to the meso-dyad-rich PNIPAM (Figure 2a), but also the chains’ overlap during contact is much more scarce for the atactic system, as compared to the other stereoisomer.

A description of the cross-link domain can be obtained from the data of Table 1. For the isotactic-rich system at 283 K, the single junction involves pairs of adjacent residues for each chain, as shown by <LJ_mean_> values, namely the inter-chain crosslink domain contains, on average, four polymer residues. Moreover, according to <NJ> values, the isotactic-rich PNIPAM forms a higher number of junctions per chain as compared to the atactic polymer. For the atactic model at 283 K, crosslinks with one residue per chain are more frequently detected.

Nakano et al. [13] reported the experimentally determined phase diagram for an aqueous solution of isotactic-rich PNIPAM with meso dyads content of 64% and average degree of polymerization of 900. At low temperatures, the polymer solution is thermodynamically stable, but it undergoes a sol-to-gel transition with increasing temperature in the investigated concentration range of 1.8%–6.0% (*w*/*w*). The sol-to-gel transition temperature decreases with the increase of concentration and it is about 296 K and 283 K at the concentrations of 2.0% and 6.0% (*w*/*w*), respectively. The isotactic-rich PNIPAM of this simulation work has an insufficient meso dyads content to form a gel phase. However, by extrapolating the information on the junction topology of Table 1, we can make the hypothesis that, in the hydrogel phase at 1.8% (*w*/*w*), similar to the polymer concentration of the simulations, the most frequent cross-links are 4-fold and involve at least four PNIPAM residues.

The concept that stereoregularity supports interchain association induces the consideration of the “*n*-cluster” model of De Gennes [27], suitable to explain the type II behaviour of the phase separation of atactic PNIPAM aqueous solutions above the LCST [28]. According to this model, attractive interactions lead to stable clusters of *n *> 2 monomers while binary monomer–monomer interactions remain repulsive. In a stereoregular chain, structurally ordered chain regions are more probable as compared to the atactic chain. These regions could be both more suitable for inter-chain matching and responsible for a less negative entropy variation in the association. By comparing these considerations with simulation results, it is noteworthy that 1–1 residue crosslinks between A and B chains are mainly observed for the atactic system, whilst junctions involving a higher number of adjacent residues are found for the isotactic-rich polymer, where the chains’ association is predominant.

### 2.2. Above the LCST

At 323 K, the aqueous solutions of both PNIPAM stereoisomers modelled in this work are not stable and a clouding behaviour is experimentally detected [22]. Accordingly, the expected result from simulations is the coil-to-globule transition and the aggregation of the chain, irrespective of stereochemistry. A remarkable increase of interchain contacts, Figure 2b, and a decrease of the overall radius of gyration, Figure 3b, are detected during the simulations at 323 K, indicating the shrinking of the polymer ensemble. In the last 90 ns trajectory, the differences between the atactic and isotactic-rich PNIPAM can be considered as negligible for these properties. Figure 4 displays the reduction of the polymer sasa at 323 K, which is higher as compared to that detected at 283 K. The difference between the sasa values of the two stereoisomers ensembles is much smaller as compared to that observed below the LCST.

**Figure 8:**
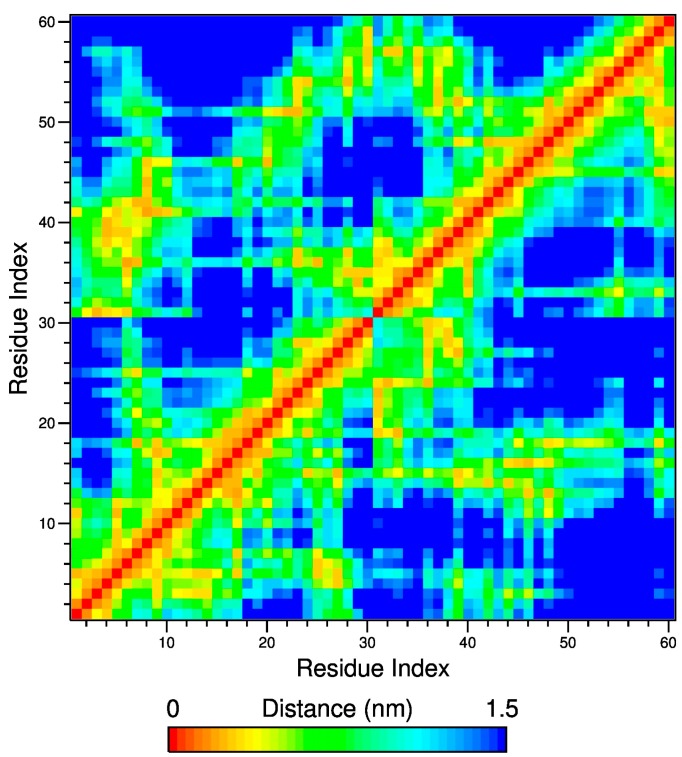
Inter-residues contact map at 323 K in the interval 200–202 ns. The top-left and bottom-right regions across the diagonal refer to atactic and isotactic-rich PNIPAM, respectively.

**Figure 9:**
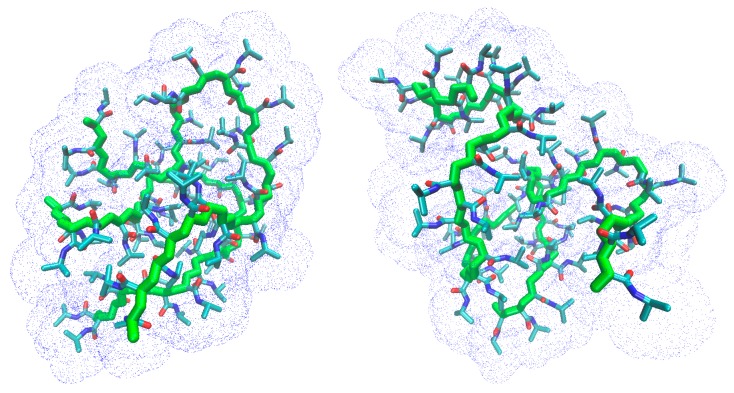
Snapshot at 202 ns of the trajectory of the atactic (**left**) and isotactic-rich (**right**) PNIPAM ensemble at 323 K. Water and hydrogen atoms are omitted. The blue dots display the solvent accessible surface.

The analysis of the matrixes of the average minimum distance between the polymer residues allows to distinguish between intra- and inter-chain contacts. Figure 8 displays this matrix for both the atactic and isotactic-rich, calculated at 323 K in the 200–202 ns trajectory interval and the corresponding snapshots are shown in Figure 9.

The inter-residue distance maps do not highlight relevant differences in both the intra- and inter-molecular association for these stereoisomers at 323 K, as compared to those observed at 283 K. The same inference comes from the analysis of the structural parameters of the interchain junctions, reported in Table 2. According to <NR> and <LJ_mean_> values of Table 2, more than one-third of residues of the 30-mer are involved in inter-chain contacts, forming several junctions of about two or three adjacent repeating units.

The extrapolation of simulation findings to the temperature-induced volume phase transition (VPT) of atactic PNIPAM chemically cross-linked microgels allows for a rough estimate of the swelling ratio, V_belowVPTT_/V_aboveVPTT_, V being the particle volume, in such systems. This estimate is applicable to micro-hydrogels with a moderate degree of cross-linking, namely with a chain length between adjacent junctions similar to the DP of these models. In these conditions, microgels typically have a water content of about 90% *w*/*w* in the swollen state. By assuming V_aboveVPTT_ as equal to the average volume of the atactic PNIPAM aggregate at 323 K, calculated by the radius of gyration of Figure 3b, and estimating V_belowVPTT_ as equal to the volume of the aqueous solution containing two atactic PNIPAM 30-mers at the 10% *w*/*w* polymer concentration, the resulting swelling ratio is equal to 12. This value compares well with experimental findings obtained for non-stereocontrolled PNIPAM microgels [29].

## 3. Conclusions

This simulation study focussed on the differences in the association features of PNIPAM chains as a function of the meso dyads content. The solution behaviour of our models, which were designed to be strictly comparable with experimentally characterized PNIPAM stereoisomers [22], highlights an increased association ability of the isotactic-rich polymer below the LCST. This result, in agreement with the findings obtained in the experiments, confirms that a relatively small variation of stereochemistry can cause large effects in the phase behaviour of PNIPAM in aqueous environment. Above the LCST, interchain and intrachain interactions contribute to a similar extent to the formation of a collapsed polymer assembly. In these conditions, the influence of tacticity on the aggregate structure is negligible.

The present work aimed to detect the characteristics of the pair interaction between polymer chains, occurring in a concentration regime immediately below the C*. This contribution represents the first step toward the atomistic detailed modelling of stereocontrolled PNIPAM microgels.

## 4. Computing Details 

Linear stereoisomers of 30 PNIPAM residues with meso dyad contents of 45% and 59%, mimicking the atactic and isotactic-rich PNIPAM, respectively, were built by assuming a Bernoullian distribution of isotactic and syndiotactic units. The dyads sequence of the stereoisomers is reported in Table 3.

The backbone dihedral angles of the polymer chain in the starting structure were set to values corresponding to states of minimum conformational energy for the dyads composing the stereoisomer [30,31]. A pair of equal stereoisomers was placed at the largest distance in a 9 × 9 × 9 nm^3^ box, maximizing also the distance with periodic images. An energy minimization in vacuo with tolerance of 10 kJ·mol^−1^·nm^−1^ was carried out; then, about 22,700 water molecules were added and a further minimization of energy with tolerance of 100 kJ·mol^−1^·nm^−1^ was performed. The structure obtained was used as the initial configuration of the simulations at both temperatures. Such models of the atactic and isotactic-rich PNIPAM solution have a polymer content of 1.6% (*wt*/*wt*), close to the chain overlap concentration, C* [22].

The MD simulations were performed using the force field OPLS-AA [32] with the modifications of Siu et al. [33] to describe the polymer, and the TIP4P/2005 model for water. In these conditions, the highly diluted solutions of the syndiotactic PNIPAM 30-mer and of the stereoisomers of the present work were successfully modelled [17,34]. Trajectories were calculated in the NPT ensemble for 210 ns both at 283 and 323 K. The leapfrog integration algorithm [35] with a 2-fs time step, cubic periodic boundary conditions and minimum image convention were used. The linear constraint solver (LINCS) procedure was applied to constrain the length of bonds involving H atoms [36]. The temperature was controlled with the velocity rescaling thermostat coupling algorithm, with a time constant of 0.1 ps [37]. The pressure was maintained at 1 atm by the Parrinello-Rahman approach, using a time constant of 2 ps [38,39]. Electrostatic interactions were treated by the smooth particle-mesh Ewald method [40], the cutoff of nonbonded interactions being set to 1 nm. The last 90 ns of trajectory were considered for analysis, sampling one frame every 5 ps. The trajectory acquisition and analysis were carried out within the GROMACS software environment (version 5.0.4) [41,42], and the graphic visualization was done using the molecular viewer software package VMD [43]. The analysis of the contacts between atoms of different chains was performed with the cutoff distances: 0.38 nm for N–O; 0.50 nm for CE_CF–CE_CF; 0.62 nm for CE_CF–CI; 0.73 nm for CI–CI. These values are the first minimum distance of the corresponding interchain radial distribution functions. The contact was taken into account when the interatomic distance was equal or lower than the cutoff value. The sasa of PNIPAM was evaluated using a spherical probe with radius of 0.14 nm and the values of van der Waals radii of the work of Bondi [44,45].

## Figures and Tables

**Table 1:** End-to-end distance of chains and features of the interchain junctions at 283 K.

Stereoisomer	<r> (nm)	<NR> ^1,2^	<NJ> ^1,2^	<LJ_mean_> ^1,2^
Atactic	3.1 ± 0.5 (A) 3.0 ± 0.7 (B)	3 ± 2 (A) 2 ± 1 (B)	3 ± 2 (A) 1.4 ± 0.6 (B)	1.2 ± 0.4 (A) 1.6 ± 0.6 (B)
Isotactic-rich	3 ± 1 (A) 1.7 ± 0.7 (B)	8 ± 3 (A) 10 ± 3 (B)	4 ± 1 (A) 5 ± 1 (B)	1.9 ± 0.6 (A) 2.0 ± 0.6 (B)

Analysis over the 120–210 ns trajectory interval. Errors estimated by standard deviation. ^1^ The capital letters A and B indicate the chain. ^2^ Average over the configurations where at least one contact is detected.

**Table 2:** Features of the interchain junctions at 323 K.

Stereoisomer	<NR> ^1,2^	<NJ> ^1,2^	<LJ_mean_> ^1,2^
Atactic	13 ± 4 (A) 12 ± 3 (B)	5 ± 1 (A) 6 ± 2 (B)	2.5 ± 0.8 (A) 1.9 ± 0.5 (B)
Isotactic-rich	12 ± 2 (A) 11 ± 2 (B)	6 ± 1 (A) 7 ± 1 (B)	2.1 ± 0.6 (A) 1.5 ± 0.2 (B)

Analysis over the 120–210 ns trajectory interval. Errors estimated by standard deviation. ^1^ The capital letters A and B indicate the chain. ^2^ Average over the configurations where at least one contact is detected.

**Table 3:** Sequence of the racemo and meso dyads of the stereoisomers.

Meso Dyads Content (%)	Dyads Sequence ^1^
45	rrrmmrmrrrrmmrrmmmmrmmrmrmrrr
59	rmmrrmrrmmrrmmmmrmmrrmmmrrmmm

^1^ r = racemo dyad; m = meso dyad.
